# News and Events of the Week

**Published:** 1911-09-30

**Authors:** 


### NEWS AND EVENTS OF THE WEEK.

The directorate of the Austrian railways has recently determined upon an interesting experiment which may possibly find imitators. As the patients going to Carlsbad and the other Bohemian watering-places are numerous, the directorate has decided that on two days a week physicians shall travel on the expresses going to these towns. On each train a doctor will hold consultations in a reserved compartment easily recognised from outside. This physician will, however, only be at the disposition of those taken suddenly ill while actually travelling. The Ministerial decree regulating this innovation ordains that only those doctors will be eligible for appointment who are able to speak German, French, and Polish. A small dispensary containing all emergency drugs will be put at the doctor's service in the reserved compartment. This year the innovation is on its trial two days a week only—one day on trains going to, and another on those coming from, Carlsbad.

The International Sanitary Conference, which is to meet in Paris on October 10, will number among its members representatives from forty States instead of from twenty-three, as at the last Conference, held in 1903. The meeting is both scientific and diplomatic in character, and inquiries will be made into modifications which, as the result of knowledge gained in the course of recent epidemics, should be introduced into present conventions regarding the prevention of outbreaks of plague and cholera.

Mr. R. F. Higgin, M.B., B.S. Lond., has been appointed Junior Resident Medical Officer at the Manchester Children's Hospital, Pendlebury.

